# Identification and Analysis of An Epigenetically Regulated Five-lncRNA Signature Associated With Outcome and Chemotherapy Response in Ovarian Cancer

**DOI:** 10.3389/fcell.2021.644940

**Published:** 2021-02-23

**Authors:** Hao Yang, Lin Gao, Meiling Zhang, Ning Ning, Yan Wang, Di Wu, Xiaomei Li

**Affiliations:** ^1^Department of Radiation Oncology, Inner Mongolia Cancer Hospital and The Affiliated People’s Hospital of Inner Mongolia Medical University, Hohhot, China; ^2^Institute for Endemic Fluorosis Control, Center for Endemic Disease Control, Chinese Center for Disease Control and Prevention, Harbin Medical University, Harbin, China; ^3^Department of Obstetrics and Gynecology, The First Affiliated Hospital of Harbin Medical University, Harbin, China; ^4^Department of Pathology, Harbin Medical University Cancer Hospital, Harbin, China

**Keywords:** ovarian cancer, long non-coding RNAs, epigenetic modification, chemotherapy response, signature

## Abstract

The deregulation of long non-coding RNAs (lncRNAs) by epigenetic alterations has been implicated in cancer initiation and progression. However, the epigenetically regulated lncRNAs and their association with clinical outcome and therapeutic response in ovarian cancer (OV) remain poorly investigated. This study performed an integrative analysis of DNA methylation data and transcriptome data and identified 419 lncRNAs as potential epigenetically regulated lncRNAs. Using machine-learning and multivariate Cox regression analysis methods, we identified and developed an epigenetically regulated lncRNA expression signature (EpiLncRNASig) consisting of five lncRNAs from the list of 17 epigenetically regulated lncRNAs significantly associated with outcome. The EpiLncRNASig could stratify patients into high-risk groups and low-risk groups with significantly different survival and chemotherapy response in different patient cohorts. Multivariate Cox regression analyses, after adjusted by other clinical features and treatment response, demonstrated the independence of the DEpiLncSig in predicting survival. Functional analysis for relevant protein-coding genes of the DEpiLncSig indicated enrichment of known immune-related or cancer-related biological pathways. Taken together, our study not only provides a promising prognostic biomarker for predicting outcome and chemotherapy response but also will improve our understanding of lncRNA epigenetic regulation mechanisms in OV.

## Introduction

Ovarian cancer (OV) is one of the most lethal gynecologic cancers and is the eighth leading cause of cancer-related deaths in women (Coburn et al., 2017; Momenimovahed et al., 2019). According to the American Cancer Society, approximately 21,750 new cases and 13,940 estimated deaths occur in 2020 in the United States (Siegel et al., 2020). Surgery followed by chemo-based treatments (platinum-based or taxane-based) is the first-line treatment, and approximately 80% of OV patients initially respond to treatment. However, most patients with advanced disease still experienced recurrent disease after a period of chemotherapy, which has led to poor outcomes with five survival rates of less than 50%, even if significant advances have been made in surgical and chemo-based treatments for OV patients. Therefore, it is critical to identify reliable and useful biomarkers for improving outcomes of OV patients.

Increasing efforts in studying molecular omics have improved our understanding of the molecular mechanisms of OV carcinogenesis and progression and contributed to the identification and development of novel molecular biomarkers and specific therapies (Cancer Genome Atlas Research Network, 2011; Lu et al., 2014; Clifford et al., 2018; Li et al., 2020). Molecular profiles have been extensively investigated and characterized during the past years, leading to the identification of a number of dysregulated molecules associated with development, progression, recurrence, metastasis, and therapeutic response of OV (Adib et al., 2004; Barrett et al., 2015; Sallinen et al., 2019; Wang et al., 2019; Zhao H. et al., 2020). Long non-coding RNAs (lncRNAs) are a significant class of non-coding RNAs (ncRNAs) that have been discovered in the last 10 years. The accumulated evidence has shown that lncRNAs are essential regulators in gene expression networks and performed a variety of functional roles in almost all biological processes through diverse mechanisms at transcriptional, post-transcriptional, and epigenetic levels (Mercer et al., 2009; Marchese et al., 2017; Yao et al., 2019). Dysregulated lncRNAs have been observed in various cancers and have also been implicated as novel biomarkers in cancer diagnosis, prognosis, and therapeutic response (Zhou et al., 2016a, 2020; Hosseini et al., 2017; Huo et al., 2017; Tian et al., 2018; Bao et al., 2020; Sun et al., 2020). Previous studies have reported that the expression of some lncRNAs can be regulated by epigenetic modification similar to protein-coding genes (Zhao et al., 2015; Wang et al., 2018; Li et al., 2019). However, the epigenetically regulated lncRNAs and their association with clinical outcome and therapeutic response in OV remain poorly investigated.

In this study, we performed an integrative analysis of DNA methylation data and transcriptome data to identify epigenetically regulated lncRNAs associated with OV carcinogenesis. Together with clinical data, we further investigated the clinical value of these epigenetically regulated lncRNAs in predicting outcome and chemotherapy response. Finally, we used the relevant genes of epigenetically regulated lncRNAs to infer their potential functions.

## Materials and Methods

### Acquisition and Analysis of DNA Methylation Data for OV Patients

DNA methylation data profiled by Illumina 27k methylation array for 583 OV tumor tissues and 12 non-cancer tissues were obtained from the UCSC Xena Browser^1^. Then probes were filtered and prepossessed as follows: (i) SNP-enriched probes were removed; (ii) those probes with missing values in more than 10% samples were removed; and (iii) imputation was performed to replace missing values by calculating the median methylation level for each sample across all CpG sites. Finally, 24793 CpG probes were kept for further analysis. Differential methylation analysis on the site level between OV tumor tissues and non-cancer tissues was conducted using the limma package. Differentially methylated CpG sites were determined based on FDR adjusted *p*-value < 0.05 and absolute mean methylation difference >0.3.

### Acquisition and Analysis of lncRNA Data of OV Patient

RNA-seq data of OV tumor tissues and non-cancer tissues were obtained from the UCSC Xena Browser (see text footnote 1). A total of 14,614 were obtained from the RNA-seq data based on GENCODE annotations^2^. The epigenetically regulated lncRNAs were identified by measuring the association between lncRNA expression and CpG levels through the Pearson correlation coefficient. Those lncRNAs correlated with CpG sites with | r| > 0.4 and *p* < 0.001 were considered as epigenetically regulated lncRNAs (DEpiLncRNAs). Hierarchical clustering analysis was conducted using the R package “pheatmap” with “ward.D2” method.

### Development of an Epigenetically Regulated Five-lncRNA Signature (EpiLncRNASig)

Univariate Cox regression analysis of DEpiLncRNAs with OS was performed to identify candidate DEpiLncRNAs with prognostic roles. Using a bidirectional elimination strategy, the stepwise regression was performed to select optimal lncRNA biomarkers from the list of candidate prognostic DEpiLncRNAs by an automatic procedure. Specifically, at each step, we added variables whose inclusion gave the most significant improvement and removed variables whose exclusion gave the most insignificant deterioration to the quality of the prediction model, which is assessed by the Akaike information criterion (AIC). Then, we repeated this process until none of them improved the model to a statistically significant extent (Zhou et al., 2020). Then a prognostic lncRNA-focused score model (EpiLncRNASig) was constructed based on the linear combination of the expression of optimal DEpiLncRNAs biomarkers, weighted by their coefficients from the multivariate regression analysis as in previous studies (Bao et al., 2020, 2021; Sun et al., 2020).

### Statistical Analysis

Univariate and multivariate Cox regression analyses were performed on the individual clinical variables with and without the EpiLncRNASig. Hazard ratios (HR) and 95% confidence intervals (CI) were calculated. Kaplan-Meier survival plots and log-rank tests were applied to compare the differences in survival time between different patient groups using the R package “survival.” All statistical analyses were performed using R Statistical Software (version 3.6.3).

### Function Enrichment Analysis

The association between expression levels of lncRNAs and mRNAs was measured by calculating the Pearson correlation coefficient, and the top 100 mRNAs were considered as lncRNA-related mRNAs. Then function enrichment analysis of Gene Ontology (GO) and the Kyoto Encyclopedia of Genes and Genomes (KEGG) was performed to infer possible functional roles of lncRNA biomarkers using the R package “clusterProfiler” (Yu et al., 2012). GO terms or KEGG pathways with adjusted *p*-value < 0.05 were considered to be significantly enriched.

## Results

### Identification of Dysregulated Epigenetically Regulated lncRNAs (DEpiLncRNAs) Associated With OV Development

**FIGURE 1:**
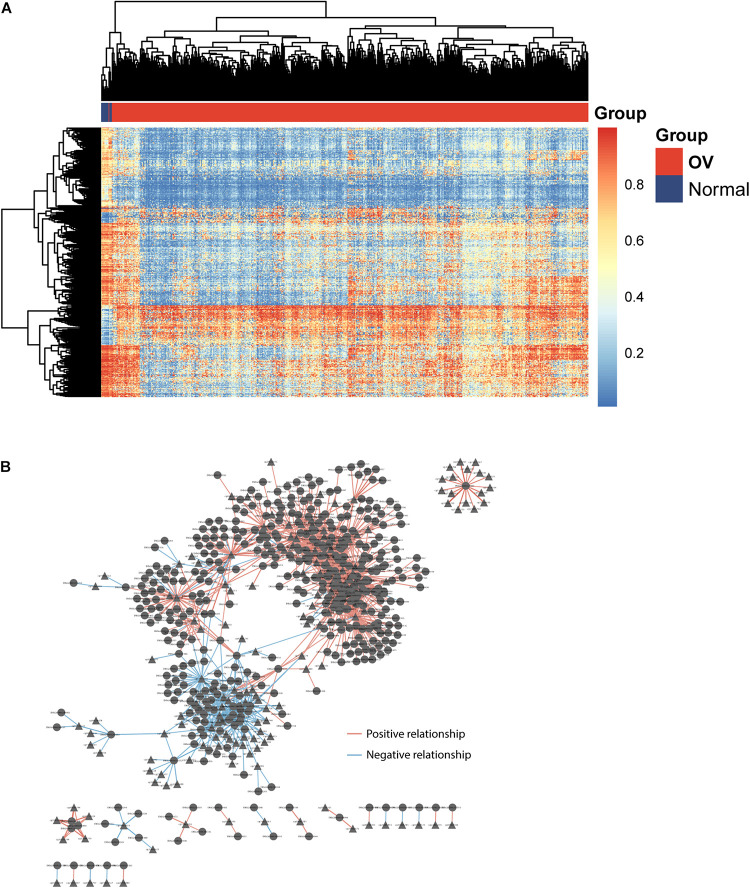
Identification of dysregulated epigenetically regulated lncRNAs (DEpiLncRNAs) associated with OV development. **(A)** Heatmap of differentially methylated sites could discriminate between OV patients with and healthy controls. **(B)** Interaction network of dysregulated epigenetically regulated lncRNAs and CpG.

To identify potential GpG sites associated with OV development, we performed compared analysis for GpG levels between OV tumor tissues and non-cancer tissues and identified 605 differentially methylated sites (FDR adjusted *p*-value < 0.05 and absolute mean methylation difference >0.3) using the R package “RnBeads.” Hierarchical clustering analysis showed that these 605 differentially methylated sites could discriminate between OV patients with and healthy controls, as shown in Figure 1A. To identify DEpiLncRNAs in OV, we calculated the Pearson correlation coefficient to evaluate the association between lncRNA expression and CpG levels and identified 1,497 lncRNA-CpG pairs, including 419 lncRNAs, which were defined as DEpiLncRNAs. The network between lncRNA and CpG sites were visualized using the Cytoscape software (Figure 1B).

### Development of an Epigenetically Regulated lncRNA Signature (DEpiLncSig) for Predicting the Outcome

**FIGURE 2:**
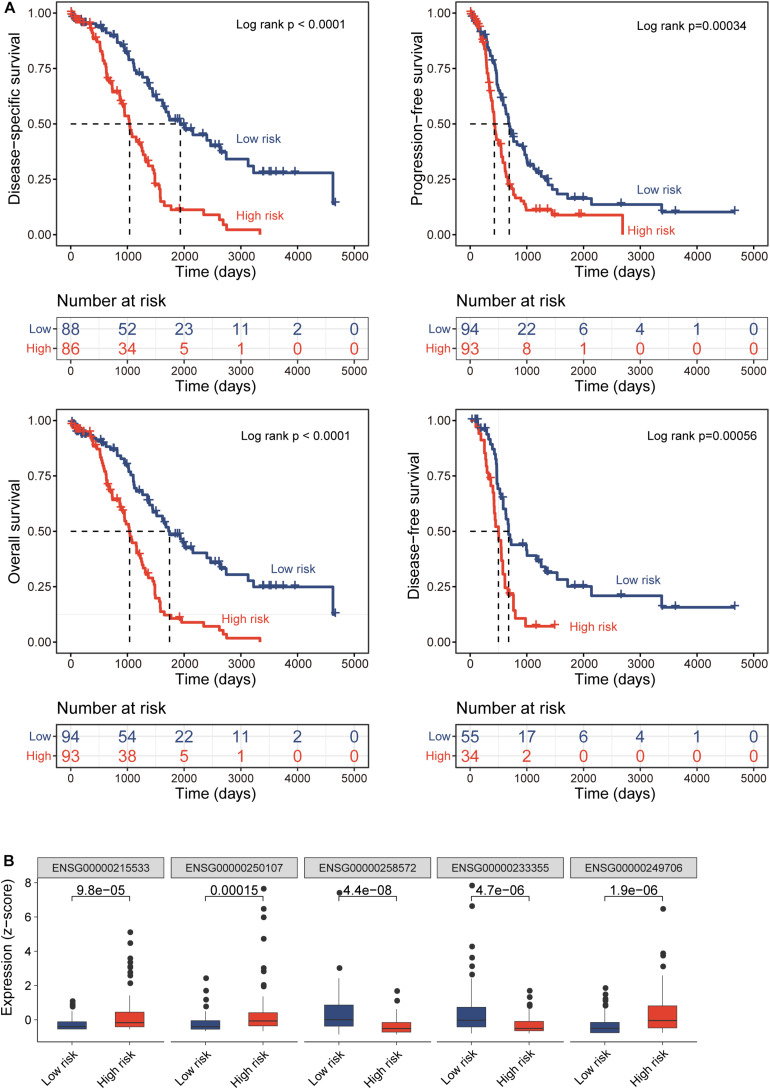
Prognostic performance of epigenetically regulated lncRNA signature (DEpiLncSig) in the discovery cohort. **(A)** Kaplan-Meier survival curves of survival times between the high-risk group and low-risk group. **(B)** Boxplots of expression levels of prognostic lncRNAs in the high-risk group and low-risk group, respectively.

All TCGA-OV patients with survival information were split equally into the discovery cohort (*n* = 187) and validation cohort (*n* = 186). We first conducted the univariate analysis for 419 lncRNAs with OS and identified 17 lncRNAs that were significantly associated with OS (*p* < 0.01). Then we used a stepwise regression model and identified five optimal lncRNA biomarkers from the list of 17 lncRNAs significantly associated with OS (Table 1). Of them, three lncRNAs (*LINC00189*, *CACNA1G-AS1*, and *AC105384.1*) seem to be risk factors, and the remaining two lncRNAs (*AL133467.1* and *CHRM3-AS2*) are protective factors. The five optimal lncRNA biomarkers and their CpGs were listed in Supplementary Table 1. Finally, we performed multivariate Cox regression analysis for five optimal lncRNA biomarkers and constructed a lncRNA-based risk score model based on the linear combination of the expression of five optimal lncRNA biomarkers, weighted by their coefficients from the multivariate regression analysis as follows: EpiLncRNASig = (0.197)^∗^expression (*LINC00189*) + (0.167)^∗^ expression (*CACNA1G-AS1*) + (−0.432)^∗^ expression (*AL133467.1*) + (−0.374)^∗^ expression (*CHRM3-AS2*) + (0.395)^∗^ expression (*AC105384.1*). Each patient in the discovery cohort was assigned a risk score and was subsequently defined as high-risk with higher EpiLncRNASig or low-risk with lower EpiLncRNASig according to the median value of the risk score (0.0538). Survival analysis showed that patients in the low-risk group have significantly improved OS (median time 1,742 days) compared to those in the high-risk group (median time 1,039 days) (log-rank test *p* < 0.0001) (Figure 2A). The 3- and 5-year survival rates of patients in the low-risk group are 75 and 48%, respectively, whereas the corresponding rates are 45 and 11%, respectively, in the high-risk group. Furthermore, there were significant differences in disease-free survival (median time 678 vs. 500 days), disease-specific survival (median time 1,933 vs. 1,039 days), and progression-free survival (median time 690 vs. 427 days) (Figure 2A). As shown in Figure 2B, three lncRNAs (*LINC00189*, *CACNA1G-AS1*, and *AC105384.1*) revealed significantly higher expression in the high-risk group compared to those in the low-risk group, and two lncRNAs (*AL133467.1* and *CHRM3-AS2*) are significantly upregulated in the low-risk group and down-regulated in the high-risk group (Figure 2B).

**TABLE 1:** Detailed information of lncRNAs in the DEpiLncSig.

Ensembl id	Gene name	Genomic location	HR^a^	*p*-value^a^
ENSG00000215533	LINC00189	Chr21: 29,193,480–29,288,205(+)	1.33 (1.11–1.59)	0.0016
ENSG00000250107	CACNA1G-AS1	Chr17: 50,556,207–50,562,108(−)	1.28 (1.11–1.48)	0.00082
ENSG00000258572	AL133467.1	Chr14: 95,516,136–95,517,911(+)	0.65 (0.5–0.83)	0.00062
ENSG00000233355	CHRM3-AS2	Chr1: 239,703,381–239,770,130(–)	0.7 (0.54–0.9)	0.0054
ENSG00000249706	AC105384.1	Chr4: 53,899,871–53,916,835(+)	1.34 (1.11–1.61)	0.002

*^*a*^derived from univariate Cox regression analysis.*

### Further Validation of the DEpiLncSig in the Independent Patient Cohort

**FIGURE 3:**
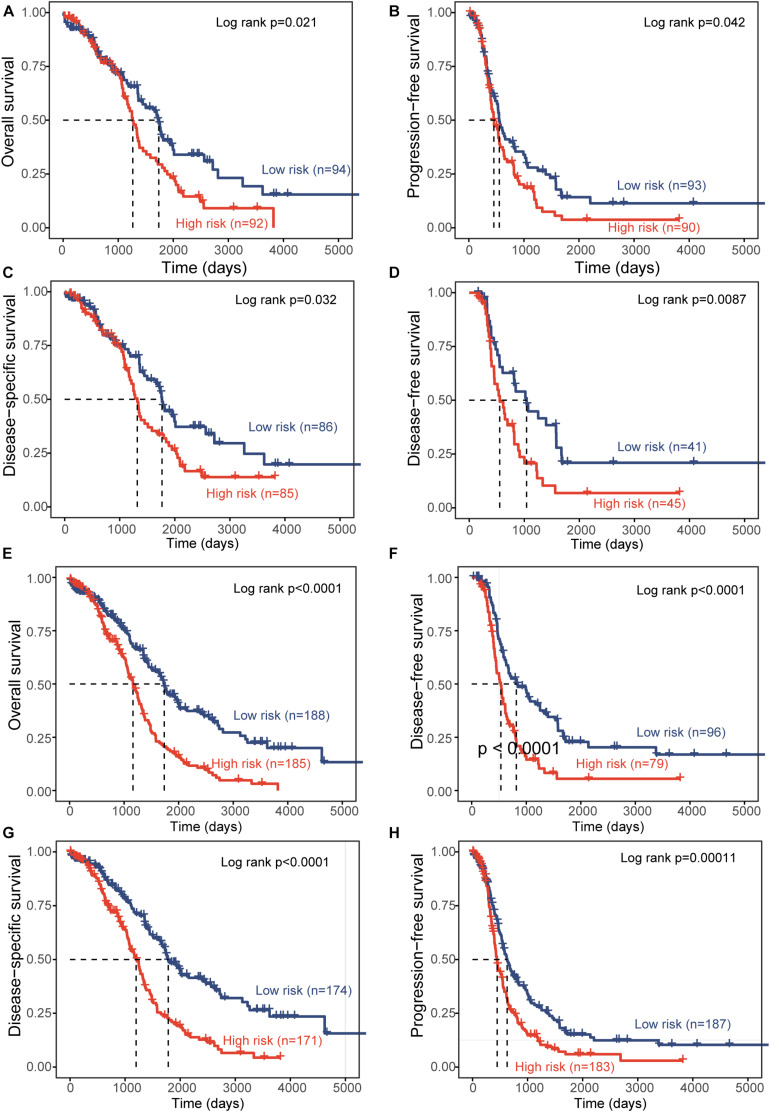
Independent validation of prognostic performance of epigenetically regulated lncRNA signature. Kaplan-Meier survival curves between the high-risk group and low-risk group for overall survival **(A)**, progression-free survival **(B)**, disease-specific survival **(C)**, and disease-free survival **(D)** in the independent patient cohort. Kaplan-Meier survival curves between the high-risk group and low-risk group for overall survival **(E)**, disease-free survival **(F)**, disease-specific survival **(G)**, and progression-free survival **(H)** in the entire TCGA-OV patient cohort.

To test the robustness of the DEpiLncSig, the DEpiLncSig was applied to 186 OV patients in the validation cohort. The DEpiLncSig classified 186 OV patients of the validation cohort into the high-risk group (*n* = 92) and low-risk group (*n* = 94) according to the risk cutoff derived from the discovery cohort. As observed in the training group, the OS time of the low-risk group patients (median 1,736 days) was significantly better than that of high-risk group patients (median 1,264 days) (log-rank test *p* = 0.021) (Figure 3A). The 3- and 5-year survival rates of patients in the low-risk group were 70 and 41%, respectively, whereas the corresponding rates are 62 and 28% in the high-risk group, respectively. Furthermore, the high-risk patients have significantly shorter disease-free survival (median time 554 vs. 1,042 days, log-rank test *p* = 0.0087), disease-specific survival (median time 1,319 vs. 1,767 days, log-rank test *p* = 0.032), and progression-free survival (median time 454 vs. 553 days, log-rank test *p* = 0.042) compared to those in the low-risk group (Figures 3B–D). When the DEpiLncSig was further tested in the entire TCGA-OV patients, all TCGA-OV patients were divided into the high-risk group and low-risk group with significantly different overall survival (median time 1,163 vs. 1,736 days, log-rank test *p* < 0.0001), disease-free survival (median time 535 vs. 818 days, log-rank test *p* < 0.0001), disease-specific survival (median time 1,199 vs. 1,784 days, log-rank test *p* < 0.0001) and progression-free survival (median time 447 vs. 628 days, log-rank test *p* = 0.00011) (Figures 3E–H). These results demonstrated the stable and reliable prognostic performance of the DEpiLncSig in predicting the outcome of OV patients.

### The Prognostic Performance of the DEpiLncSig Is Independent of Other Clinical Features

To test whether the prognostic performance of the DEpiLncSig is independent of other clinical features, we conducted univariate and multivariate Cox regression analyses for the individual clinical variables (age, stage, grade, and treatment response) with and without the EpiLncRNASig. As shown in Table 2, the EpiLncRNASig and treatment response were all significantly associated with overall survival in the univariate and multivariate analysis in the discovery cohort. In the validation cohort, although the EpiLncRNASig (HR = 1.56, 95% CI = 1.07–2.28, *p* = 0.022), age (HR = 1.59, 95% CI = 1.09–2.31, *p* = 0.015) and treatment response (HR = 0.2, 95% CI = 0.13–0.33, *p* = 4.50E-11) were all significantly associated with overall survival in the univariate analysis, only the EpiLncRNASig (HR = 1.71, 95% CI = 1.09–2.67, *p* = 0.019) and treatment response (HR = 0.19, 95% CI = 0.12–0.31, *p* = 3.80E-11) were significant in the multivariate analysis. In the entire TCGA-OV cohort, univariate analysis showed that the EpiLncRNASig (HR = 2.09, 95% CI = 1.6–2.74, *p* = 6.80E-08), age (HR = 1.35, 95% CI = 1.04–1.75, *p* = 0.022), and treatment response (HR = 0.23, 95% CI = 0.17–0.32, *p* = 1.70E-18) were significantly associated with overall survival in the univariate analysis. However, the EpiLncRNASig (HR = 2.21, 95% CI = 1.61–3.05, *p* = 1.20E-06) still maintained a significant association with overall survival when after adjusted by other clinical features (Table 2). Results from multivariate Cox regression analyses suggested that the prognostic performance of the DEpiLncSig is independent of other clinical features and treatment response.

**TABLE 2:** Univariate and Multivariate Cox regression analysis with overall survival in different patient cohorts.

Variables		Univariate analysis			Multivariate analysis		
			
		HR	95% CI	*p*-value	HR	95% CI	*p*-value
**Discovery cohort**							
Score	High vs. Low	2.83	1.93–4.16	1.10E-07	2.97	1.86–4.75	5.60E-06
Age	>60 vs. ≤60	1.14	0.8–1.64	0.47	1.08	0.71–1.65	0.72
Stage	III/IV vs. I/II	3.11	0.77–12.62	0.11	16310394	0-Inf	1
Grade	3/4 vs. 1/2	1.13	0.67–1.89	0.65	1.11	0.62–1.98	0.73
Treatment response	CR vs. non-CR	0.26	0.16–0.41	5.70E-09	0.33	0.2–0.53	3.80E-06
**Validation cohort**							
Score	High vs. Low	1.56	1.07–2.28	0.022	1.71	1.09–2.67	0.019
Age	>60 vs. ≤60	1.59	1.09–2.31	0.015	1.41	0.91–2.17	0.12
Stage	III/IV vs. I/II	1.44	0.53–3.92	0.48	1.08	0.33–3.56	0.89
Grade	3/4 vs. 1/2	1.42	0.74–2.72	0.3	1.47	0.7–3.07	0.31
Treatment response	CR vs. non-CR	0.2	0.13–0.33	4.50E-11	0.19	0.12–0.31	3.80E-11
**TCGA-OV cohort**							
Score	High vs. Low	2.09	1.6–2.74	6.80E-08	2.21	1.61–3.05	1.20E-06
Age	>60 vs. ≤60	1.35	1.04–1.75	0.022	1.25	0.92–1.69	0.15
Stage	III/IV vs. I/II	2.01	0.89–4.53	0.092	1.64	0.51–5.28	0.41
Grade	3/4 vs. 1/2	1.23	0.82–1.84	0.32	1.3	0.83–2.05	0.25
Treatment response	CR vs. non-CR	0.23	0.17–0.32	1.70E-18	0.24	0.17–0.33	1.10E-16

### Association of the EpiLncRNASig With Chemotherapy Response

**FIGURE 4:**
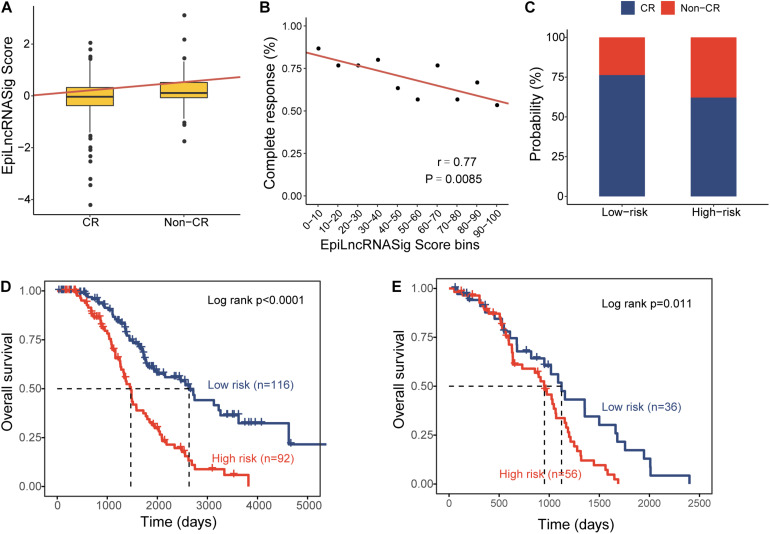
Association of the EpiLncRNASig with chemotherapy response. **(A)** Boxplots of EpiLncRNASig scores for patients with CR and non-CR. **(B)** Correlation of the EpiLncRNASig with complete response. **(C)** The percentage of patients achieving CR in the high-risk group and low-risk group. Kaplan-Meier survival curves of overall survival times between the high-risk group and low-risk group for patients with CR **(D)** and non-CR **(E)**.

To further examine the association of the EpiLncRNASig with a chemotherapy response, we first compared the distribution of the risk score of the EpiLncRNASig for patients with complete response (CR) and non-CR, and we found that patients with CR have significantly lower risk scores than those with non-CR (Wilcoxon rank-sum test *p* = 0.0011) (Figure 4A). We then assessed the relationship between the EpiLncRNASig and the likelihood of CR by plotting the percentage of OV patients achieving CR as a function of the risk score and found that there was a significant association between risk score and the likelihood of CR (Pearson correlation coefficient *r* = −0.77, *p* = 0.0085) (Figure 4B). As shown in Figure 4C, patients with low EpiLncRNASig seem to have a higher likelihood of CR than those with high EpiLncRNASig. To further examine whether the EpiLncRNASig is predictive for both patients achieving CR and non-CR, we performed a stratification analysis. The results of the stratification analysis showed that the EpiLncRNASig could further subdivide patients achieving CR into the high-risk group and low-risk group with significantly different survival times (log-rank *p* < 0.0001) (Figure 4D). Similar results were observed for patients achieving non-CR (log-rank *p* = 0.011) (Figure 4E).

### *In silico* Analysis for lncRNA Biomarker Function

**FIGURE 5:**
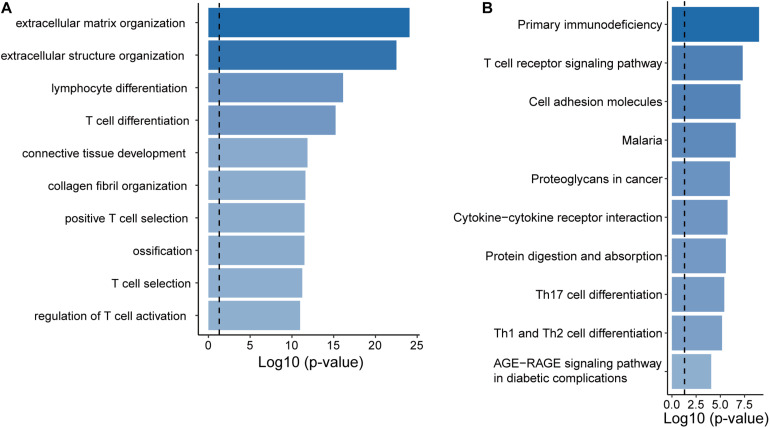
Functional prediction of the EpiLncRNASig. **(A)** Functional enrichment analysis for GO terms. **(B)** Functional enrichment analysis for KEGG pathways.

To infer potential biological roles of these epigenetically regulated lncRNA biomarkers, we calculated the Pearson correlation coefficient between expression levels of lncRNAs and mRNAs, and selected top 100 mRNAs were considered as lncRNA-related mRNAs (Supplementary Table 2). Then we performed GO and KEGG function enrichment analysis for 471 lncRNA-related mRNAs. Results of GO analysis showed that lncRNA-related mRNAs were enriched in the extracellular matrix organization, connective tissue development, lymphocyte differentiation, and T cell differentiation and activation (Figure 5A). Results of KEGG analysis showed that lncRNA-related mRNAs were enriched in immune-related or cancer-related biological pathways, including primary immunodeficiency, cell adhesion molecules, proteoglycans in cancer, cytoline-cytokine receptor interaction and the T cell receptor signaling pathway, Th17 cell differentiation, Th1, and Th2 cell differentiation (Figure 5B).

## Discussion

Accumulating evidence has shown that altered molecular profiles are an indispensable laboratory tool to improve cancer diagnosis, prognosis and therapeutic response (Malone et al., 2020), and overcome the limitations of typical clinical and imaging characteristics owing to the genetic and molecular heterogeneity of cancers. During the past years, lncRNAs have become a rising star in the field of biomarker research and were extensively studied and characterized in various cancers. Aberrant lncRNA expressions have also been observed in OV development, progression, recurrence, metastasis, and therapeutic response, indicating the potential roles as biomarkers for precision medicine of OV. Several lncRNA-focused expression signatures have been proposed for the prediction and monitoring of disease status, prognosis, and chemotherapeutic sensitivity. For example, Pan et al. (2020) proposed two lncRNA signatures to predict the prognosis and efficiency of chemotherapy. Zhou et al. (2016b) constructed progression-related lncRNA-associated ceRNA networks based on the “ceRNA hypothesis” and subsequently identified a 10-lncRNA signature associated with the outcome of OV patients. However, further investigation is needed to identify epigenetically regulated lncRNAs and explore their potential function and clinical application in OV.

For the above purpose, we performed an integrative analysis of DNA methylation data and transcriptome data and identified 419 lncRNAs as potential epigenetically regulated lncRNAs. These predicted epigenetically regulated lncRNAs in this study would provide an available resource for studying the interplay between lncRNAs and epigenetic regulation. A recent study performed by Li et al. (2019) has characterized the crosstalk between DNA methylation and lncRNA expression in thyroid Cancer (THCA) and methylation-driven 5-lncRNA-based signature (EpiLncPM) with potential clinical application in predicting the prognosis of THCA. Therefore, to further investigate whether epigenetically regulated lncRNAs identified here also have potential clinical value, we performed univariate Cox regression analysis with OS and found that only 17 of 419 epigenetically regulated lncRNAs identified here were significantly associated with patients’ outcome. By using machine-learning and multivariate Cox regression analysis methods, we identified and developed an epigenetically regulated lncRNA expression signature (EpiLncRNASig) consisting of five lncRNAs from the list of 17 epigenetically regulated lncRNAs associated with outcome. Furthermore, we tested and validated these in different patient cohorts, demonstrating a similar effective predictive performance in predicting OS. Moreover, multivariate Cox regression analyses after adjusted by other clinical features and treatment response demonstrated the independence of the DEpiLncSig in predicting OS. Further examination of the association of the EpiLncRNASig with chemotherapy response provided evidence supporting that the DEpiLncSig is not only a prognostic factor but may also be an indicator for chemotherapy response.

Compared to the huge number of lncRNAs identified and recorded in the public database, the number of functionally well-characterized lncRNAs is relatively small. Among five lncRNAs in the EpiLncRNASig, several lncRNAs have been well studied in some cancers. For example, *LINC00189* has been reported to be associated with several cancers, including ovarian cancer, cervical cancer (Zhang et al., 2020), clear cell renal cell carcinoma (Xu et al., 2020), and urinary bladder cancer (Zhang et al., 2016). By analyzing *CACNA1G-AS1* expression levels in 122 pairs of non-small cell lung cancer (NSCLC) and normal tissue samples as well as in NSCLC cell lines, Yu et al. (2018) identified CACNA1G-AS1 as an oncogene to promote cell migration, invasion, and epithelial-mesenchymal transition via regulating *HNRNPA2B1*. A recent study performed by Wei revealed higher expression of *CACNA1G-AS1* in colorectal cancer (CRC) tissues compared to adjacent normal tissues and found that *CACNA1G-AS1* was able to enhance proliferative and invasive abilities of CRC cells by downregulating p53 via forming a carcinogenic complex with *EZH2* (Wei et al., 2020). Another recent study has reported that lncRNA *CACNA1G-AS1* can act as competing endogenous RNAs (ceRNAs) to regulate miR-205 expression, and it promotes proliferation and invasion in human keloid fibroblasts (Zhao X. et al., 2020). To further gain novel insights into the functional roles of the EpiLncRNASig in OV, we performed functional enrichment analysis for relevant protein-coding genes of epigenetically regulated lncRNAs by considering their co-expression relation relationships and found that the EpiLncRNASig was involved in known immune-related or cancer-related biological pathways.

In conclusion, we reported and provided a knowledge base of novel epigenetically regulated lncRNAs in OV, which will improve our understanding of lncRNA epigenetic regulation mechanisms in OV. Finally, we proposed an epigenetically regulated five-lncRNA signature as promising prognostic biomarkers for predicting outcome and chemotherapy response with further validation in prospective cohort studies.

## Data Availability Statement

Publicly available datasets were analyzed in this study. This data can be found here: DNA methylation and RNA-seq data of OV patients were obtained from the UCSC Xena Browser (https://xena.ucsc.edu/) under the cohort accession: GDC TCGA Ovarian Cancer (OV).

## Author Contributions

XL and DW conceived and designed the experiments. HY, LG, MZ, NN, and YW performed the experiments and analyzed the data. DW and XL wrote the manuscript. All authors read and approved the final manuscript.

## Conflict of Interest

The authors declare that the research was conducted in the absence of any commercial or financial relationships that could be construed as a potential conflict of interest.

## Footnotes

1 https://xena.ucsc.edu/

2 https://www.gencodegenes.org/
